# *Trichomonas vaginalis* adherence phenotypes and extracellular vesicles impact parasite survival in a novel *in vivo* model of pathogenesis

**DOI:** 10.1371/journal.pntd.0011693

**Published:** 2023-10-23

**Authors:** Brenda M. Molgora, Sandip Kumar Mukherjee, Sharon Baumel-Alterzon, Fernanda M. Santiago, Katherine A. Muratore, Anthony E. Sisk, Frances Mercer, Patricia J. Johnson

**Affiliations:** 1 Molecular Biology Institute, University of California, Los Angeles, Los Angeles, California, United States of America; 2 Department of Microbiology, Immunology and Molecular Genetics, University of California, Los Angeles, Los Angeles, California, United States of America; 3 Laboratory of Immunoparasitology “Dr. Mário Endsfeldz Camargo,” Department of Immunology, Institute of Biomedical Sciences, Federal University of Uberlândia, Uberlândia, Brazil; 4 Department of Pathology, David Geffen School of Medicine, University of California, Los Angeles, Los Angeles, California, United States of America; 5 Department of Biological Sciences, California State Polytechnic University, Pomona, Pomona, California, United States of America; Federal University of Minas Gerais: Universidade Federal de Minas Gerais, BRAZIL

## Abstract

*Trichomonas vaginalis* is a human infective parasite responsible for trichomoniasis–the most common, non-viral, sexually transmitted infection worldwide. *T*. *vaginalis* resides exclusively in the urogenital tract of both men and women. In women, *T*. *vaginalis* has been found colonizing the cervix and vaginal tract while in men it has been identified in the upper and lower urogenital tract and in secreted fluids such as semen, urethral discharge, urine, and prostatic fluid. Despite the over 270 million cases of trichomoniasis annually worldwide, *T*. *vaginalis* continues to be a highly neglected organism and thus poorly studied. Here we have developed a male mouse model for studying *T*. *vaginalis* pathogenesis *in vivo* by delivering parasites into the murine urogenital tract (MUT) via transurethral catheterization. Parasite burden was assessed *ex-vivo* using a nanoluciferase-based gene expression assay which allowed quantification of parasites pre- and post-inoculation. Using this model and read-out approach, we show that *T*. *vaginalis* can be found within MUT tissue up to 72 hrs post-inoculation. Furthermore, we also demonstrate that parasites that exhibit increased parasite adherence *in vitro* also have higher parasite burden in mice *in vivo*. These data provide evidence that parasite adherence to host cells aids in parasite persistence *in vivo* and molecular determinants found to correlate with host cell adherence *in vitro* are applicable to infection *in vivo*. Finally, we show that co-inoculation of *T*. *vaginalis* extracellular vesicles (*Tv*EVs) and parasites results in higher parasite burden *in vivo*. These findings confirm our previous *in vitro*-based predictions that *Tv*EVs assist the parasite in colonizing the host. The establishment of this pathogenesis model for *T*. *vaginalis* sets the stage for identifying and examining parasite factors that contribute to and influence infection outcomes.

## Author summary

*Trichomonas vaginalis* is a human infective parasite that is responsible for most common, non-viral, sexually transmitted infection worldwide. Despite the over 270 million cases of T. vaginalis infection reported annually worldwide, the parasite continues to be a highly neglected pathogen and thus poorly studied. Despite several attempts to establish an animal model to study *T*. *vaginalis* infection, reliable infection models are lacking. As a result, our understanding of parasitic features that contribute to establishing and maintaining an infection have been determined by growing cultures of parasites and human host cells in the laboratory and testing parasite:host cell interactions in test tubes. Here we describe the development of a mouse model for studying *T*. *vaginalis* infection in an animal. We demonstrate that two features of the parasite that affect parasite: host cell interaction in test tubes: (1) its adherence properties and (2) the extracellular vesicles it releases that are taken up by host cells also impact parasite survival in this novel, mice model. The establishment of this animal model for *T*. *vaginalis* sets the stage for identifying and examining parasite factors that contribute to and influence infection outcomes.

## Introduction

*Trichomonas vaginalis* (*Tv*) is an extracellular, eukaryotic protist and the causative agent of trichomoniasis–the most common, non-viral, sexually transmitted infection worldwide [1]. Worldwide prevalence of trichomoniasis surpasses that of gonorrhea, syphilis, and chlamydia combined with reports of roughly 156 million new cases worldwide and over 276 million cases annually [2–4]. Clinical manifestations of trichomoniasis range from inflammation of the male and female urogenital tracts to more severe complications such as infertility, an increased risk of human immunodeficiency virus (HIV) transmission and adverse pregnancy outcomes in women such as preterm delivery and low infant birth weight [5–12]. While it is assumed that the parasite can infect both sexes with equal frequency [13,14], the asymptomatic nature of infection in men coupled with the unreliable diagnostic testing for men has resulted in an underestimation in infection numbers [15–18]. As men are primarily asymptomatic, they can act as vectors for parasite transmission by passing *Tv* on to their partners [12–15,19]. Additionally, *Tv* has been associated with an increased risk of aggressive prostate cancer, the second leading cause of cancer death among men in the United States [20–24].

To establish infection, *Tv* adheres to the epithelial lining of the urogenital tract by transitioning from a free-swimming ovoid cell into its adherent amoeboid form [25,26]. It is thought that the level of parasite binding to host cells seen *in vitro* mimics the outcome of infection *in vivo* [27], but this has correlation has yet to be investigated. *Tv* adherence to the host occurs through the use of multiple adherence factors. While some of these factors have been identified, such as lipoglycans (LG) [28,29], BspA-like proteins [30], a rhomboid serine protease [31], a cadherin-like protein [32], and a host glycosaminoglycan-interacting protein [33], no single player can fully recapitulate maximal adherence. Therefore, this signifies that parasite adherence to the host epithelium is multifactorial, with additional players still unknown [34,35].

Despite numerous attempts to establish an *in vivo* vaginal mouse model for the study of trichomoniasis [36–40], no reliable model has been established. Variability in the murine vaginal microbiota [41], hormonal variations during the menstrual cycle of women and female mice, as well as differences in vaginal pH, are thought to contribute to the difficulty of establishing a robust *Tv* infection in female mice [39,41]. A male murine model for the study of prostatitis caused by *Tv* has been reported using Wistar rats [42], suggesting that male mice might also be successfully infected by *Tv*. However, this study primarily focused on pathological changes and failed to provide a method for quantification of parasite burden. Furthermore, there is a smaller repertoire of genetically modified or knock-out rats compared to mice, which would hinder studying host:*Tv* interactions, and the use of rats as an animal model can prove to be more expensive long term.

Here we have established an *in vivo T*. *vaginalis* pathogenesis model using the male mouse urogenital tract (MUT). Using nanoluciferase as a readout for parasite burden, we determined that *Tv* can be found up to 72 hrs post-inoculation in MUT tissues. To our knowledge this is the first study which employs nanoluciferase as a readout for parasite burden in tissues *ex-vivo*. Using this model, we demonstrate that *Tv* strains which are more adherent to host cells *in vitro* result in higher parasite burden *in vivo*, providing the first direct evidence that parasite adherence to host cells affects the ability of the parasite to establish infection *in vivo*. Furthermore, we also demonstrate that the presence of *Tv* extracellular vesicles (EVs) results in higher parasite burden *in vivo*, and confirm previous *in vitro*-based predictions that *Tv*EVs assist the parasite in colonizing the host [43,44].

## Methods

### Ethics statement

Research was conducted under an Institutional Animal Care and Use Committee (IACUC) approved protocol in compliance with the Animal Welfare Act, Public Health Service (PHS) Policy on Humane Care and Use of Laboratory Animals, and other Federal statutes and regulations relating to animals and experiments involving animals. The facility where this research was conducted is accredited by the Association for Assessment and Accreditation of Laboratory Animal Care (AAALAC), International and adheres to principles stated in the Guide for the Care and Use of Laboratory Animals, National Research Council, 2011. Animals meeting pre-established criteria were humanely euthanized in accordance with American Veterinary Medical Association guidelines.

### Mice

BALB/cJ male mice were obtained from The Jackson Laboratory (Bar Harbor, ME) at 8 weeks of age. All procedures were reviewed and approved by the Institutional Animal Care and Use Committee of the University of California, Los Angeles Research Safety and Animal Welfare Administration (protocol ARC 2009–063).

### Parasites and media

*T*. *vaginalis* strains MSA 1103 and LSU 160 MA and P, derived from LSU 160 [33] were used. MSA 1103 and LSU 160 are clinical isolates from St. Paul Minnesota, USA and New Orleans, Louisiana USA, respectively, and have been described in Lustig et al. 2013 [27]. LSU 160 MA (more adherent) and LSU 160 P (parental) strains were derived from LSU 160 using a novel selection method to create isogenic parasites that differ in their ability to adhere to host cells, as we previously described [33]. Briefly, a clonal population of LSU 160 parasites was passaged for eight weeks by separating parasites that bound to the culture tubes (MA) or passaging without selection to tube-bound parasites (P) daily. The MA parasites were found to be approximately 6-fold more adherent to BPH-1 host cell monolayers, relative to the P parasites [33]. Parasite strain B7RC2, described in Lustig et al. 2013 [27], was used for the isolation of extracellular vesicles (see *Extracellular vesicle (EV) isolation* below*)*. Parasites were cultured in Diamond’s modified Trypticase-yeast extract-maltose (TYM) medium supplemented with 10% horse serum (Sigma-Aldrich), 10 U/mL penicillin/10 μg/mL streptomycin (Gibco), 180 μM ferrous ammonium sulfate, and 28 μM sulfosalicylic acid [45,46] and were grown at 37°C and passaged daily. Overnight cultures of MSA 1103 and LSU 160 P were placed on ice for 10 min and vortexed for 30 sec prior to passaging to a concentration of 5 x 10^4^ cells/mL in 15 or 50 mL fresh completed media. LSU 160 MA strain was passaged as previously described [33]. Briefly, an overnight culture of LSU 160 MA was decanted to remove free-swimming parasites from the culture and replaced with fresh completed medium. The culture is then placed on ice for 10 min and passaged as described above.

### Nucleofections with the nanoluciferase expression construct

*T*. *vaginalis* parasites were nucleofected as previously described [33,47]. Briefly, 100 μg of the *T*. *vaginalis* specific plasmid, pMasterNeo [48], harboring the nanoluciferase (Nluc) gene [49] was nucleofected into MSA 1103 and LSU 160 parasites using T-cell buffer (Lonza) + D-023 program or V-kit buffer (Lonza) + X-001 program, respectively. Recovered parasites were selected with 100 μg/mL G418 (Invitrogen) 24 hrs post-nucleofection. Presence of the Nluc plasmid was confirmed via nanoluciferase activity assay described below.

### Mouse inoculations using transurethral catheterization

All experiments were performed on 10-14-week-old BALB/cJ male mice. Transurethral inoculation of the mouse MUT tissue was carried out as previously described [50] with modifications. Briefly, fluid-deprived (4 hrs) mice were anesthetized with isoflurane and catheterized using a sterile polyethylene Intramedic PE-10 catheter (BD Biosciences) lubricated with Surgilube (HR Pharmaceuticals Inc.). 1 x 10^8^ Nluc-expressing parasites resuspended in 100 μL RPMI-1640 (+ L-glutamine, + HEPES; Invitrogen) supplemented with 10 U/mL penicillin/10 μg/mL streptomycin, and 10% fetal bovine serum without G418 to avoid any toxicity G418 may have on the recipient mice and injected into the MUT. Mice were kept anesthetized for an additional 30 min post-inoculation to increase the chance of colonization and minimize parasite loss through urination. Infected animals were sacrificed at the indicated times post-inoculation. The MUT tissue (prostate glands, bladder, urethra, and seminal vesicles) were excised and processed for parasite quantification via luciferase activity, protein concentration, or tissue histology as described below.

### Tissue histology

Two mice were euthanized immediately after inoculation with 5 x 10^7^ *T*. *vaginalis* MSA 1103 parasites and the harvested MUT tissue was fixed in 10% formalin overnight at room temperature. The tissues were then rinsed for 15 min under running water and stored in 70% ethanol. Fixed tissues were embedded in paraffin, serially sectioned at a width of 5 μm, and stained with Hematoxylin & Eosin (H&E) staining. Samples were imaged using a BX43 light microscope (Olympus) and Aperio ScanScope AT Turbo Whole Slide Scanning System (Leica Biosystems) and analyzed using the Aperio ImageScope software (Leica Biosystems).

### Tissue processing

Infected mice were euthanized at respective time points and the MUT tissue was excised and placed in pre-weighed tubes containing 5 mL 1x PBS (-CaCl_2_, -MgCl_2_) and placed on ice until ready for further processing. Tubes + MUT were weighed to determine the weight of the tissue and the tissue was then minced and resuspended in 1x MUT lysis buffer (50 mM Tris-HCl, 2 mM EDTA, 0.1% Triton X-100, pH 8 + 1x HALT protease inhibitor (Thermo Fisher)) at a volume of 1 mL for every 0.5 g tissue. Lysates were then sonicated using a Sonic Dismembrator F60 (Fisher Scientific) probe sonicator on ice at 60% power for 5 cycles, 0.5 min on/off, set at setting 12 which was followed by centrifugation at 10,000 rpm at 4°C for 10 min to pellet insoluble material. Supernatant was collected and used for Nluc or Bradford assays further described below.

### Nanoluciferase activity assay

The linear dynamic range of the NanoGlo Luciferase Assay System (Promega) in *T*. *vaginalis* parasites was determined per the manufacturer’s instructions. Briefly, the NanoGlo reagent was prepared by mixing the NanoGlo substrate in NanoGlo buffer at a ratio of 1:50. Serial dilutions of *T*. *vaginalis*-Nluc parasites were prepared (ranging from 0 parasites/100 μL to 10^6^ parasites/100 μL) by diluting parasites in 1x MUT lysis buffer. 100 μL of protein dilutions were added to the wells of a 96-well, round bottom white plate (Corning) in triplicate wells and mixed with 100 μL NanoGlo reagent per well. Luminescence was read using a Synergy H1 Hybrid Multi Mode (BioTek) plate reader at 460 nm. Data was analyzed using Gen5 Microplate Reader and Imager software (BioTek) and Microsoft Excel.

Measuring luminescence of mouse MUT tissue inoculated with Nluc-expressing *T*. *vaginalis* parasites was carried out similarly with one minor difference. 100 μL of the processed MUT tissue supernatant was added to the wells of a 96-well, round bottom white plate in triplicate and mixed with 100 μL NanoGlo reagent. The samples were read and analyzed as described above.

### Bradford assay

To quantify protein concentration of the infected MUT tissue, processed supernatants were diluted 1:10 in 1x MUT lysis buffer and 5 μL of the diluents were transferred to a 96-well, flat bottom clear plate (Corning) in triplicate wells. Samples were then mixed with 200 μL 1x Bio-Rad Protein Assay (Bio-Rad) reagent. Absorbance was read using a Synergy H1 Hybrid Multi Mode plate reader at 595 nm. Absorbance values of serially diluted bovine serum albumin (BSA) done in triplicate was used to generate a standard curve. Data was analyzed using Gen5™ Microplate Reader and Imager software as well as Microsoft Excel.

### Extracellular vesicle (EV) isolation

Extracellular vesicles (EVs) were isolated from *T*. *vaginalis* strain B7RC2 as previously described [43,44] and resuspended in 100 μL PBS+1x HALT protease inhibitor. After determining the concentration of EV protein using a Pierce BCA Kit (Thermo Scientific), EVs were stored at -80°C until ready for use.

### Co-infection with EVs and parasites

Mouse inoculations using transurethral catheterization were conducted as described above. Prior to inoculating the mice, 1 x 10^8^ MSA 1103 Nluc-expressing parasites were pre-incubated with 50 μg/μL EVs for 30 min at RT. Mice were sacrificed at 0, 48 and 72 hrs post-infection and MUT tissue was processed and nanoluciferase activity assays were performed as described above. Parasite only controls were treated identically.

### Statistical analyses

Graphs were generated and statistical analyses performed using Prism7 (GraphPad) software. Two-tailed t test was used to determine significance for *in vivo* luminescence/μg protein. Repeated measures one-way ANOVA was used to determine significance in Nluc signal levels of parasites grown in the absence of G418. One-way ANOVA with Tukey’s multiple comparisons test was used to determine significance for *in vivo* luminescence/μg protein data and total parasite counts following recovery from mouse MUT tissue. Unpaired t-test with Welch’s correction was used to determine significance in *in vivo* luminescence/μg protein data normalized to 0 hr control mice. Data are expressed as ± standard deviation (± SD).

## Results

### Delivering T. vaginalis into the mouse male urogenital tract

**Fig 1:**
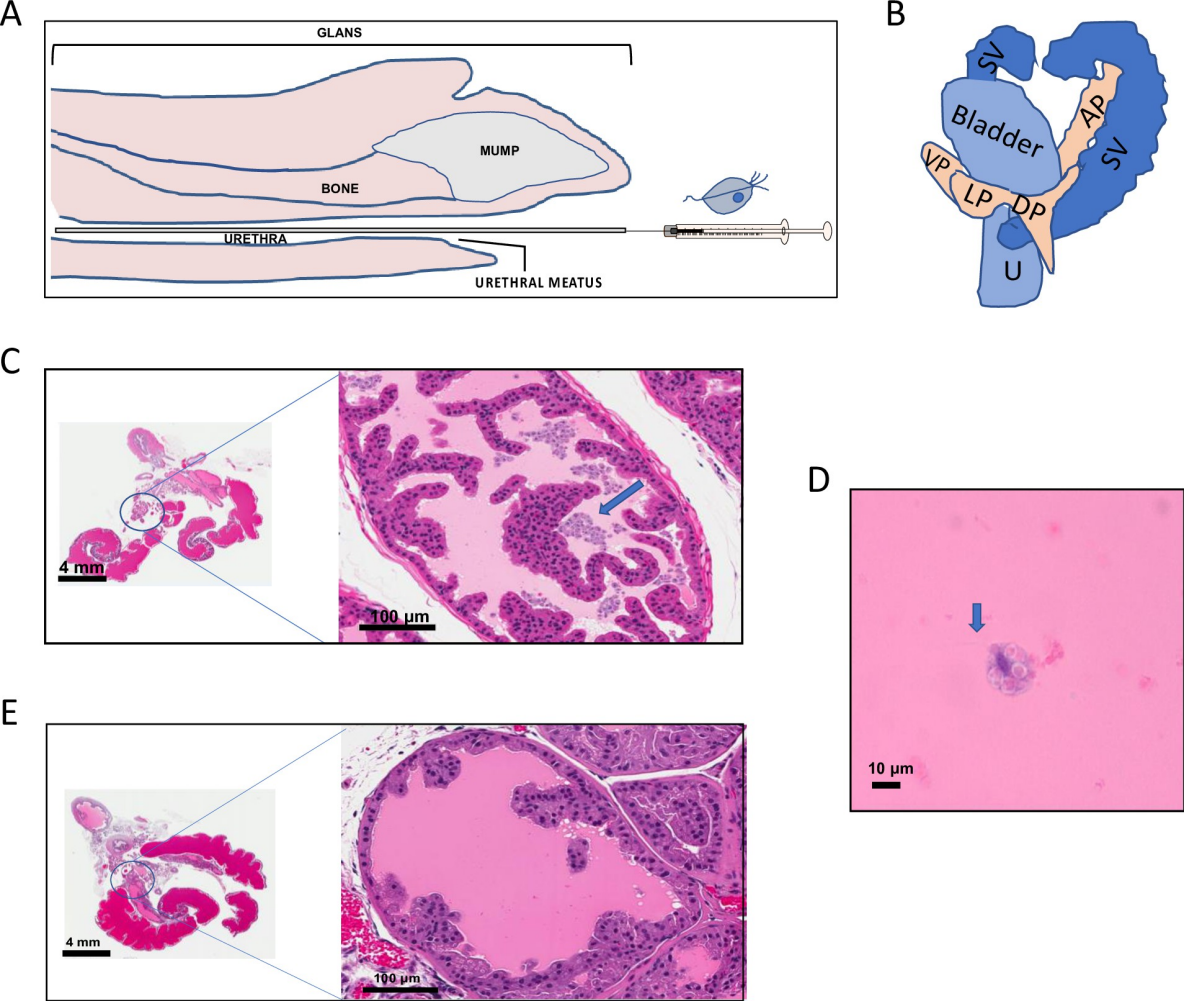
*Tv* is delivered into the mouse male urogenital tract (MUT) using urethral catheterization. (A) Diagram of catheterization and *Tv* delivery into the mouse MUT. 10^8^ Nluc expressing parasites suspended in 100 μl RPMI-1640 medium were introduced through the urethral meatus into the urethra via a syringe and polyethylene catheter. Additional mouse penile features such as the glans, penile bone, and the male urogenital mating protuberance (MUMP) are depicted. Mouse penis sketch adapted from a mid-sagittal sectioning of the adult mouse penis [74]. (B) A simplified diagram of a lateral view of the male urogenital system in adult mice modified from Cunha *et*. *al*. [75]. Parasites introduced into the mouse MUT via urethral catheterization would be delivered through the urethra (U) to the anterior (AP), dorsal (DP), lateral (LP), and ventral (VP) prostatic lobes. The bladder and seminal vesicles (SV) are also depicted. Histological validation of *Tv* delivery into the mouse MUT (C) versus the vehicle control. (E) The right panels are a 20X magnification of the left anterior prostate lobe (marked by a circle in the left panels). *T*. *vaginalis* parasites are indicated by a blue arrow. (D) 100X magnification of *Tv* parasite found in infected mouse MUT tissue. Blue arrow indicates the presence of flagella.

*T*. *vaginalis* (*Tv*) has been found in the semen, urethral discharge, urine, and prostatic fluids of infected men [15,51]. In this study, we investigated whether the mouse male urogenital tract [MUT] can serve as a tractable *in vivo* model to study *Tv* pathogenesis. Transurethral catheterization was employed to deliver *Tv* to the mouse prostate as this approach has been used to successfully introduce *Escherichia coli* [50,52–54], *Propionibacterium acnes* [55], and *Chlamydia muridarum* [56] into the mouse MUT, as well as delivery of *Tv* to the rat prostate [42]. *Tv* is introduced into the mouse MUT via a syringe and catheter which is inserted past the urethral meatus and into the urethra (Fig 1A) which flows to the bladder, seminal vesicles, and 4 lobes of the mouse prostate (Fig 1B).

To confirm *Tv* delivery into the mouse MUT via transurethral catheterization, two mice were injected with *Tv* strain MSA 1103 parasites, immediately euthanized, and the MUT tissue excised. The tissues were then paraffin embedded and examined using Hematoxylin & Eosin (H&E) staining. Stained tissues were shown to contain parasites (Fig 1C), based on the presence of cells with a pear-shaped morphology and central/anterior elongated nuclei and anterior flagella (Fig 1D). Cells with this morphology were not seen in the mock vehicle control tissue (Fig 1E). A greater number of parasites were observed within the anterior lobes likely due to their larger size and anatomy, as opposed to a preference for this location.

### Nanoluciferase as Quantifiable measure of Tv burden in vivo

**Fig 2:**
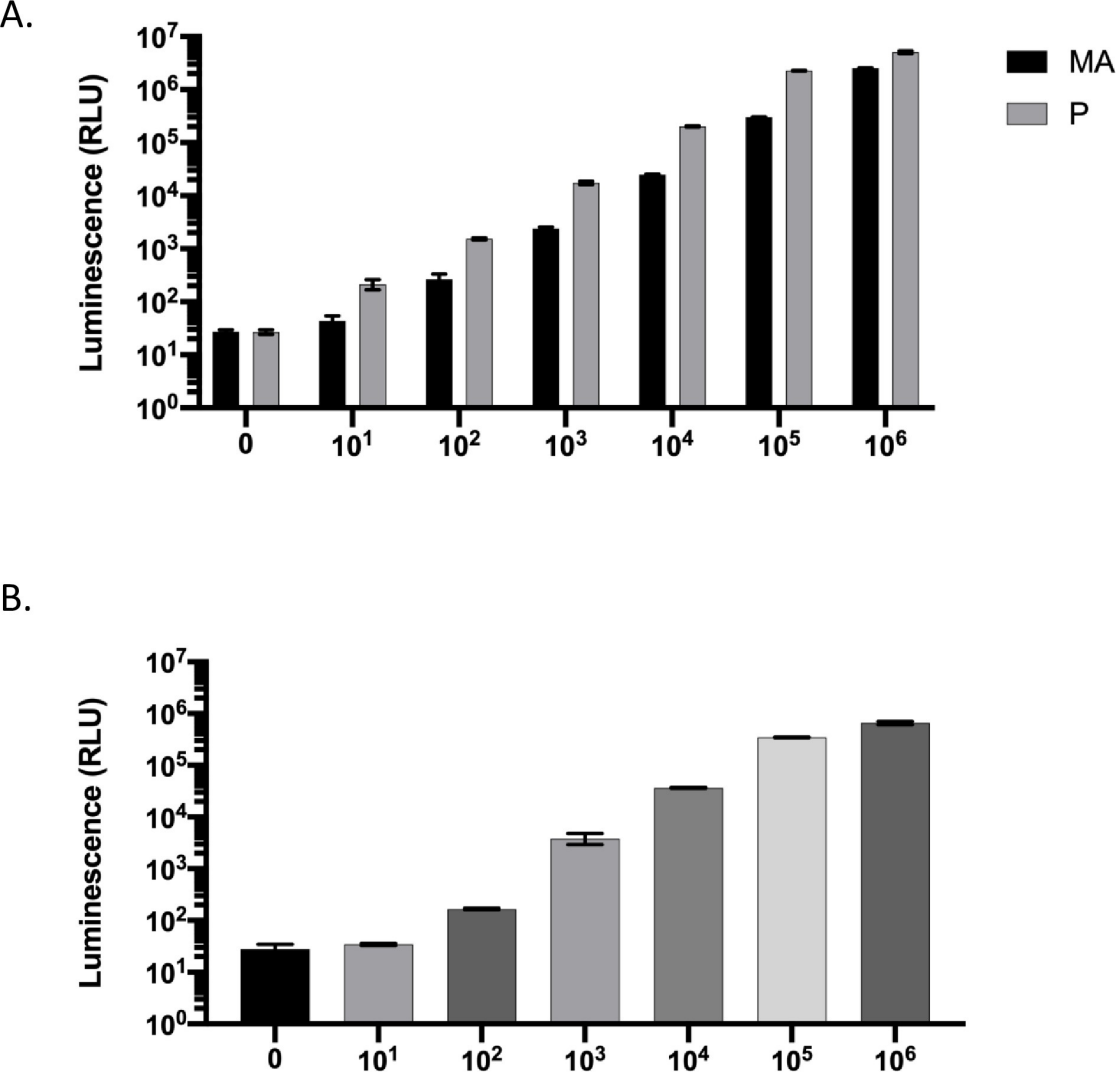
Linearity of luminescence in different Nluc-expressing *Tv* strains. *T*. *vaginalis* strains LSU 160 MA and P (A) and MSA 1103 (B) nucleofected with pMasterNeo-Nluc plasmid were measured for luminescence at varying amounts of parasites from 0 to 10^6^ parasites (as indicated on the X axis) to determine the linear range of the assay for each strain. Data shown are averages of luminescence signal from 3 technical replicates with standard deviation for each strain.

We initially examined whether available *Tv*-specific antibodies could be used to accurately quantify parasites using immunofluorescent detection with immunohistochemistry but found this to be an unreliable method due to high background levels. As a result, we developed a method which uses parasites expressing the nanoluciferase (Nluc) gene as a quantitative measure of parasite load in inoculated mouse MUT tissue. Nanoluciferase has been used to analyze infection and lifecycles *in vivo* for other eukaryotic pathogens [57,58] and has also been used in *Tv* for an *in vitro* proof-of-function approach [47]. To confirm Nluc expression and detectability as well as determine the linear range of the assay, a serial dilution of the Nluc-harboring *Tv* used in this study were assayed (Fig 2). Three variants of *Tv* strains were examined: the LSU 160 parental (P) and more adherent (MA) isogenic strains, which differ in their abilities to adhere to host cells [33], and the MSA 1132 strain. The LSU 160-P parasites displayed a notable difference in luminescence compared to the LSU 160-MA parasites (Fig 2A). The MSA 1103 parasites displayed a similar linear range to that of the LSU 160- P parasites, however the MSA 1103 maximum luminescence within the linear range was emitted by 10^5^ parasites rather than 10^6^ as seen with LSU 160-P (Fig 2B). To determine whether Nluc signals would diminish over time post-inoculation of parasites into the mouse MUT, in the absence of G418 selection of the Nluc plasmid, we grew parasites in the absence of G418 *in vitro* and measured Nluc luminescence over 72 hrs. No differences in luminescence signal were detected (S1 Data).

**Fig 3:**
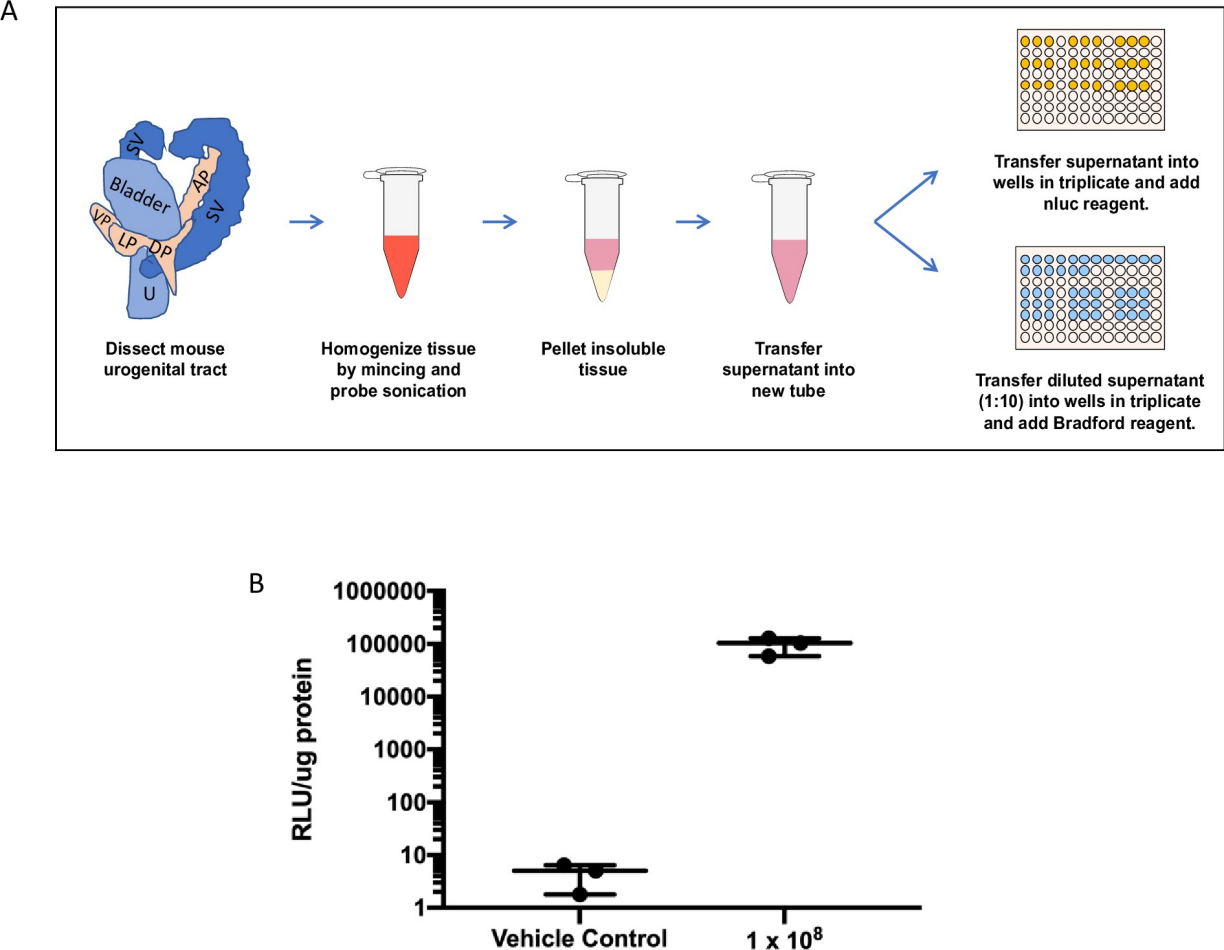
Quantifying parasite burden using nanoluciferase-expressing *Tv*. (A) Schematic of mouse MUT tissue sample preparation and quantification of *Tv* parasite burden. Excised mouse MUT tissue is finely minced and suspended in prostate lysis buffer (2 mL/mg tissue). MUT tissue then undergoes additional homogenization via probe sonication to ensure complete tissue dissociation. Insoluble material is pelleted via centrifugation and the supernatant is transferred to a new tube to further quantify luciferase activity and protein concentration using the Nano-Glo luciferase and Bradford assays, respectively. Detailed description found in materials and methods. (B) Mice were inoculated with 10^8^ parasites (n = 3) or RPMI vehicle control (n = 3) and promptly euthanized. Samples were processed and analyzed by Nluc and Bradford assays to quantify parasite load. Data is shown as average luminescence signal/μg protein of the sample.

To confirm that *Tv*-Nluc parasites would yield a detectable signal post-inoculation into the mouse MUT, we introduced 10^8^ *Tv*-Nluc parasites or RPMI medium into three mice per condition. We then harvested the MUT tissues and homogenized the tissue by mincing and probe sonication to prepare the samples for Nluc and Bradford assay analysis (Fig 3A). *T*. *vaginalis*-Nluc infected mice display a significantly higher Nluc signal (*p* = 0.0088) at 96,600 ± 20,200 RLU/μg protein compared to the vehicle control at 4.41 ± 1.37 RLU/μg protein (Fig 3B). Together, these data illustrate that *Tv* can be reproducibly delivered into the mouse MUT and that Nluc-expressing parasites can be harvested, and parasite burden measured via luciferase activity and total protein quantification.

### Quantification of Tv in the mouse urogenital tract up to 72 hours post-inoculation

**Fig 4:**
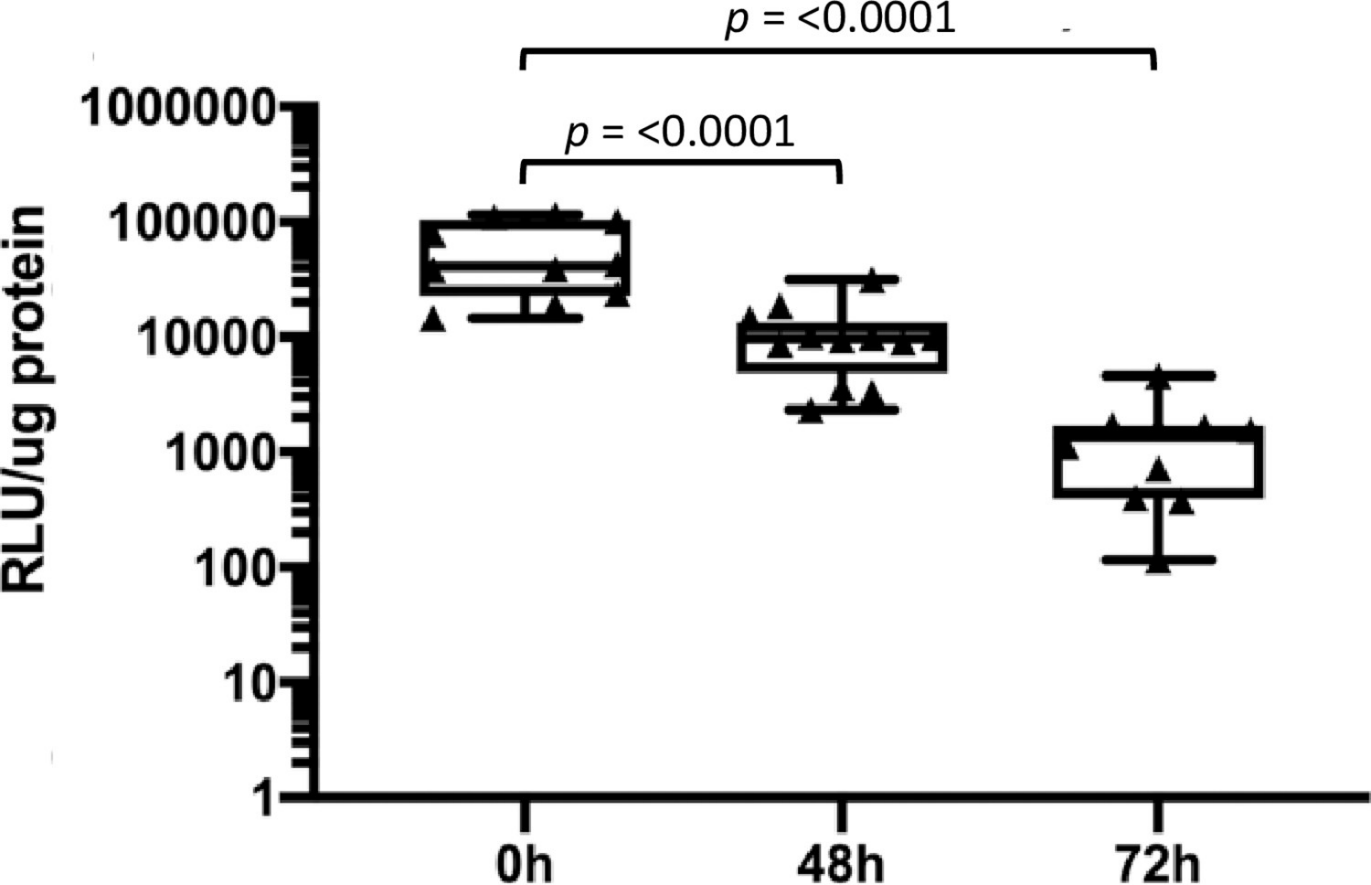
*Tv* found within the murine MUT tissue 72 hours post-inoculation. Quantification of Nluc-expressing MSA 1103 parasites in infected mouse MUT tissues 48 hrs or 72 hrs post-inoculation (as indicated on the X axis). Data are based on 12 infected mice per time point per strain and are averages of luminescence/μg protein of the sample with standard deviation. Data are representative of 3 independent experiments.

**Fig 5:**
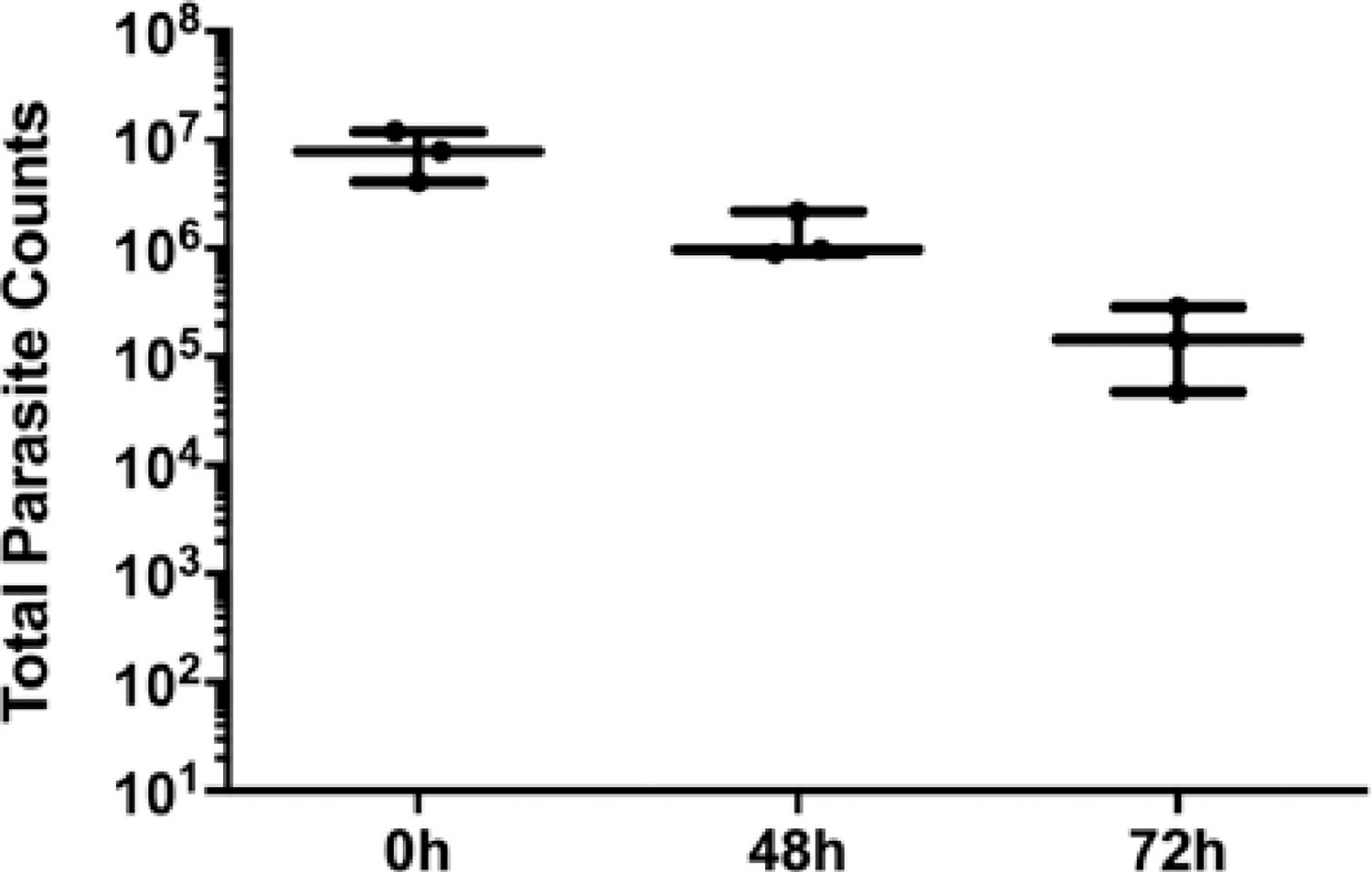
Viable parasites are recovered from mouse MUT tissue 72 hours post-inoculation. 10^8^ *Tv* parasites were introduced into the mouse urogenital tract and the infected tissue was then excised at 0 hr, 48 hrs, and 72 hrs post-inoculation (as indicated on the X axis). Tissue was minced, dissociated using Accutase cell detachment solution, resuspended in completed modified TYM media and counted via hemocytometer. Data shown are the total number of parasites per sample with standard deviation and were carried out using 3 mice per timepoint.

Having established the basis for our male mouse model, we asked whether parasites persist in the mouse MUT over time. Using *Tv* strain MSA 1103, as it has a statistically significant preference for prostate cells *in vitro* [27], we quantified parasite burden at 48 and 72 hrs post-inoculation of the Nluc-expressing *Tv*. Parasite burden decreased from 58,500 ± 12,500 RLU/ μg protein at 0 hrs to 12,500 ± 2,720 RLU/μg protein (*p* < 0.0001) at 48 hrs and 1,400 ± 404 RLU/μg protein (*p* < 0.0001) at 72 hrs (Fig 4]. Parasite burden could not be measured at time points past 72 hrs as the signal fell on or below the Nluc assay limit of detection (S2 Data). Furthermore, to confirm that viable parasites were being recovered from the MUT tissue at these time points, *Tv*-inoculated tissue was processed and resuspended in Diamond’s modified TYM media to count parasites via hemocytometer. Similar to the Nluc data, parasite numbers also decrease over time (Fig 5). This data demonstrates that Nluc signal reflects parasite numbers, as required to directly compare parasite survival in mice over a 72 hr period.

### Increased Host-Cell adherence in vitro correlates with increased parasite survival in vivo

It is well-established that *T*. *vaginalis* adherence to host cells *in vitro* varies between strains [27,59,60]. In addition, biochemical and genetic analyses indicate that these strains may differ in other properties in addition to their adherence phenotypes [47,61–64]. To measure parasite infectivity based solely on adherence ability, isogenic strains of *T*. *vaginalis* with different adherence abilities [33] (more adherent–MA; parental–P) were used to determine if *in vitro* host cell adherence is correlated with *in vivo* survival. Equivalent numbers of MA and P parasites (10^8^/100 μL) were delivered into the mouse MUTs using transurethral catheterization and parasite burden was determined 48 hrs and 72 hrs post-inoculation. Eleven to fourteen mice were analyzed for the MA strain at each time point while nine to twelve mice were used for the P strain. MUT tissues were processed, and parasites were quantified as described above.

**Fig 6:**
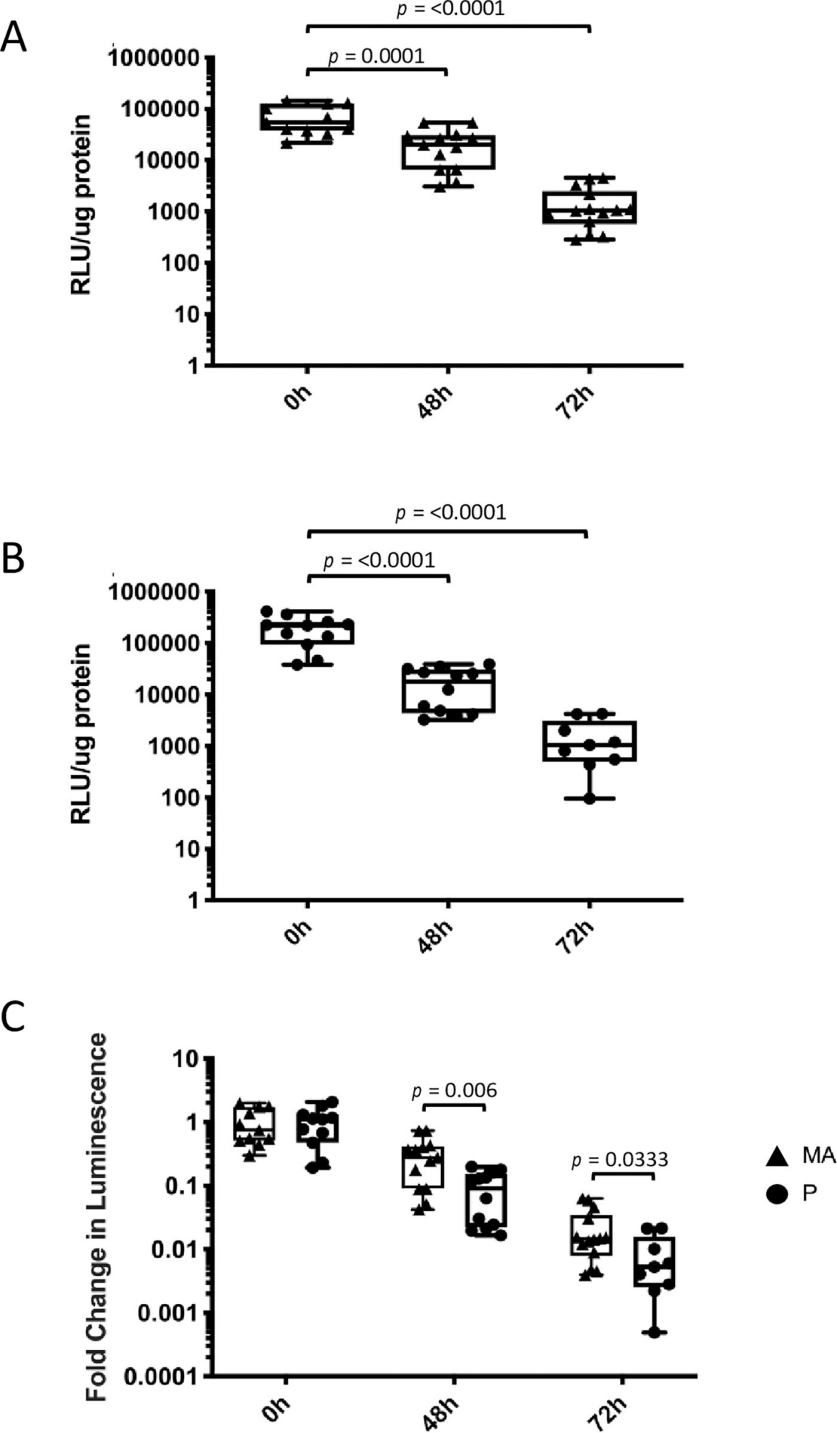
*Tv* adherence to host cells *in vitro* correlates with an increased parasite burden in the MUT. Quantification of Nluc-expressing LSU 160 more adherent (MA) (A) or parental (P) (B) parasites in mouse MUT tissues at 48 hrs and 72 hrs post-inoculation (as indicated on the X-axis). 11–14 mice were used for the MA strain at each time point. 9–12 mice were used for the P strain at each time point. Data shown are averages of luminescence/μg protein of the sample with standard deviation and are representative of 3 independent experiments. (C) Comparison of LSU160 MA (▲) and LSU160 P (●) parasite load quantifications. Data is expressed as fold change in luminescence in the mouse urogenital tract with standard deviation normalized to their respective 0h post-inoculation luminescent signals.

At 48 hrs post-inoculation, MA Nluc signal was 22,400 ± 4670 RLU/μg protein and 1,590 ± 389 RLU/μg at 72 hrs compared to 72,300 ± 13,500 RLU/μg protein seen at 0 hrs (Fig 6A). This is a maximum reduction of ~1.75 orders of magnitude in signal per μg protein between 0 hrs and 72 hrs. A similar trend was observed at 48 and 72 hrs post-inoculation for P parasites, with the signal averaging at 19,100 ± 3920 RLU/μg and 1620 ± 523 RLU/μg, respectively compared to 198,000 ± 36,200 RLU/μg protein seen at 0 hrs (Fig 6B). This is a maximum reduction in signal per μg protein of ~2 orders of magnitude between 0 hrs and 72 hrs However, taking into account that P parasites express Nluc at higher levels than MA, as seen in Fig 2A, MA and P data was normalized to their respective 0 hrs and compared to see if the enhanced *in vitro* adherence of MA [33] results in increased survival *in vivo*. At both 48 hrs and 72 hrs, MA yields significantly higher parasite burden at ~3.4 (*p* = 0.006) and ~2.7 (*p* = 0.03) times higher than P, respectively (Fig 6C). These data demonstrate that the MA parasites have higher infection burden *in vivo* than P parasites, consistent with the observed increased adherence of MA parasites, relative to P parasites, to host cells *in vitro* [33]. The higher parasite burden observed for MA parasites indicates that greater adherence to host cells facilitates parasite infectivity and maintenance *in vivo*.

### Parasite extracellular vesicles (EV) increases the survival of parasites in vivo

**Fig 7:**
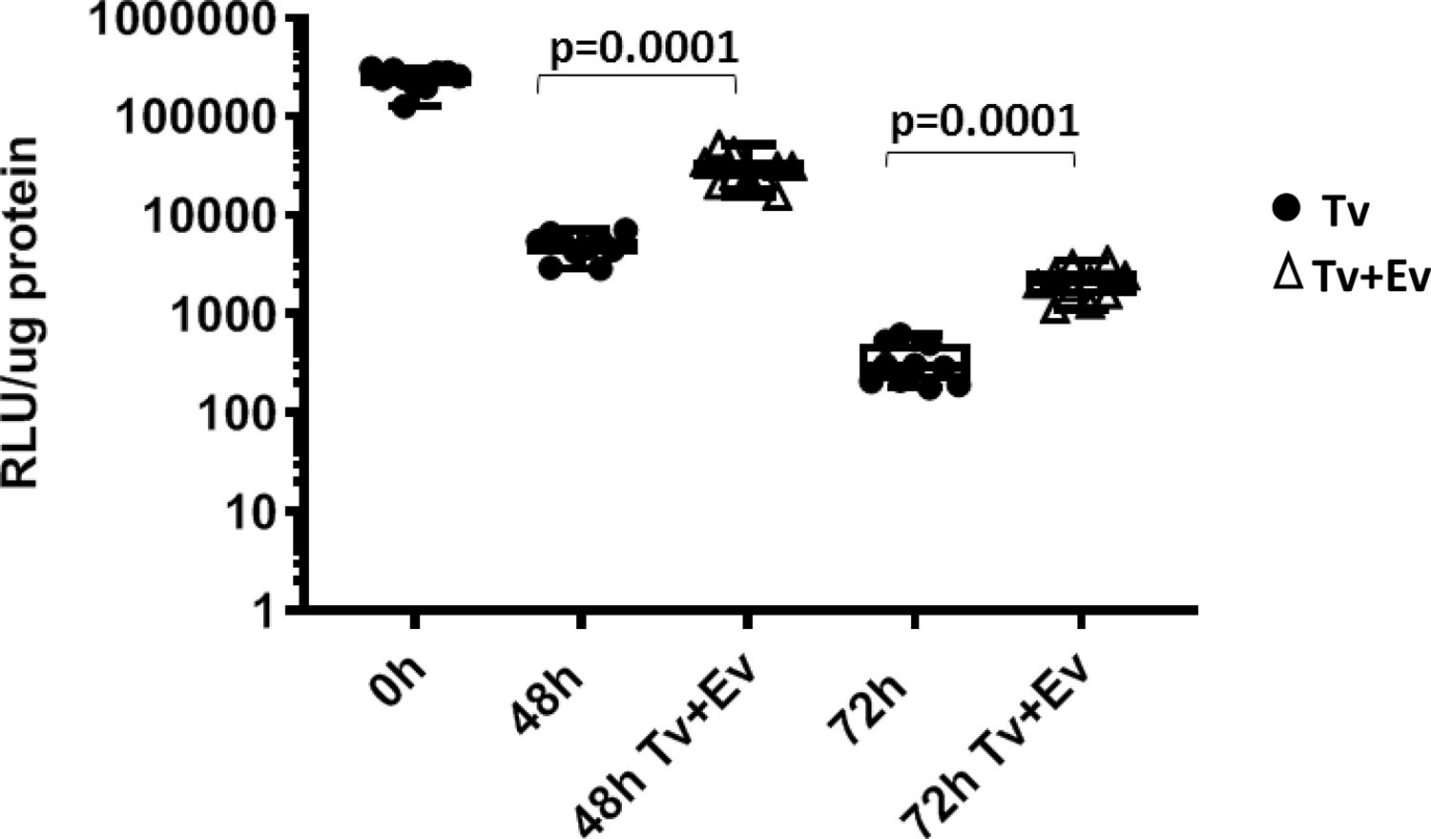
Co-inoculation of parasite secreted EVs with *Tv* increases the parasite burden in the mouse urogenital tract. Quantification of Nluc-expressing MSA 1103 parasites co-inoculated with either 50 μg/μL EVs (▢; *Tv*+EV) or parasites only (●; *Tv*) in the mouse MUT tissues at 48 and 72 hrs post-inoculation (as indicated on the X-axis). Ten mice were inoculated at each time point per condition. Data shown are averages of luminescence/μg protein of the sample with standard deviation normalized to 0h post-inoculation luminescent signals. Data are representative of 3 independent experiments.

We have demonstrated that extracellular vesicles (EVs) secreted from *Tv* are internalized by host cells [44,65] and increase the adherence of this extracellular parasite to host cells [43] *in vitro*, indicating EVs likely assist in parasite colonization of the host *in vivo*. To determine whether EVs affect the colonization and survival of parasites *in vivo*, we inoculated the mouse MUTs with equivalent amounts (1 x 10^8^) of either MSA 1103 parasites preincubated for 30 minutes with 50 μg/mL EVs or preincubated with the vehicle control, using transurethral catheterization. Parasite burden was then measured at 0, 48 and 72 hrs post-inoculation. At 48 and 72 hrs post-inoculation, MSA 1103 Nluc signal was 4887 ± 2167 RLU/μg protein and 530 ± 152 RLU/μg, respectively, from mice inoculated with parasites only, compared to 29,965 ± 18,294 RLU/μg and 2150 ± 1181 RLU/μg, respectively, from mice co-inoculated with parasites and 50 μg/mL EVs. This is a maximum increase of ~1.8 and ~1.3 orders of magnitude in signal per μg protein, respectively, in mice co-inoculated with parasites and EVs, relative to those inoculated with parasites only (Fig 7). At both 48 and 72 hrs post-inoculation, mice co-inoculated with parasites and EVs had parasite burdens at ~6.1 (*p* = 0.0001) and ~4.1 (*p* = 0.0001) times higher than mice inoculated with parasites only (Fig 7). These data demonstrate that the presence of EVs significantly increases parasite survival *in vivo*. This increased parasite survival *in vivo* in the presence of EVs is consistent with our observation that EVs increase parasite adherence to host cells *in vitro* [43,66] and likely support the colonization and survival of the parasites in the MUT.

## Discussion

We have developed a male mouse model for the study of *T*. *vaginalis* (*Tv*) pathogenesis *in vivo*. Using transurethral catheterization, the male urogenital tract (MUT) of BALB/cJ mice was inoculated with *Tv* parasites that express a nanoluciferase gene. After confirming *Tv* delivery into the mouse MUT and identifying the linear range of our Nluc assay to allow for signal comparison across different time points post-inoculation (Figs 1–3), live parasites recovered from the MUT tissue 24 hrs and 72 hrs post-inoculation were assessed (Fig 4). Using this method to quantify parasite burden in tissues *ex-vivo*, we demonstrated that parasites could be detected up to 3 days post-inoculation (Fig 5). Having established this mouse model, we tested whether *Tv* isolates with an increased adherence to host cells *in vitro* survived better in the mouse MUT *in vivo* (Fig 6) and whether the addition of parasite EVs increased parasite burden (Fig 7).

To determine whether there is a correlation between the ability of a *Tv* strain to adhere to host cells *in vitro* and survival in the mouse MUT *in vivo*, isogenic strains of *Tv* which differ in their adherence phenotypes to host cells *in vitro* [33] were examined (Fig 6). We found that the more adherent (MA) parasites isolated from inoculated MUT tissues exhibited ~3.4-fold higher signal/μg protein at 48 hrs and ~2.7-fold higher signal/μg protein at 72 hrs compared to their less adherent parental (P) counterparts (Fig 6C). These data indicate that parasite adherence to host cells *in vitro* can be correlated with survival *in vivo*. This is consistent with strains displaying higher levels of *in vitro* adherence [27] which would likely cause a higher titer and more sustained survival. *Tv* is auxotrophic for several essential nutrients and must acquire these from the host or culture media when grown in culture. Nutrient acquisition is thought to require host cell lysis [35] and it has been shown that parasites must adhere to host cells to lyse them [27]. It is notable that in a *Listeria monocytogenes* infection model, highly adherent strains were found to be more infective to the murine liver compared to lowly adherent strains [67]. These data provide the first demonstration that *Tv* adherence to the host epithelium may aid in establishing an infection *in vivo* and underscores the usefulness of using *in vitro* biochemical approaches to identify additional adherence factors which may ultimately inform design of additional therapeutics to treat infections.

We also established a correlation between the ability of parasite secreted EVs to increase the adherence of *Tv* to host cell monolayers *in vitro* [43] and increased survival of the parasite in our murine model (Fig 7). We found that the addition of EVs to parasite inoculations enhanced survival in the mice ~6.1 and ~4.1-fold, 48 and 72 hrs post-inoculation, relative to mice inoculated with parasites alone. These data illustrate for the first time that *Tv*EVs play a role in host:parasite interactions *in vivo* and confirm previous *in vitro*-based predictions that TvEVs assist the parasite in colonizing the host in vivo [43,44].

Our male mouse model allows the introduction of *Tv* in the prostate without the need of pretreatments with hormones, antibiotics, corticosteroids or *Lactobacillus*, the predominant bacteria of the human vaginal microbiome [36,68], as is necessary in mouse vaginal infection models [36,37,41,69]. Using the prostate as an infection model has allowed us to avoid issues in reproducibility and parasite titer sustainability as seen in the *Tv* vaginal models monitoring early infection which is thought to arise from the required pretreatments. The vaginal microbiome is also known to affect the outcome of vaginal infections [70,71]. The sterility of the upper male urogenital tract [72] likely contributes to our ability to infect male mice without the need to manipulate the site of infection. Furthermore, this model may be useful for probing the possible role of bacterial infection(s) in the non-sterile urethra for the establishment of *Tv* infection in the male urogenital tract. This is the first male murine model for *Tv* infection to quantify parasite survival and examine the role of parasite adherence properties and the effect of EVs on colonization and survival. Our model differs from the *Tv* rat prostatitis model [42], which was established to examine tissue pathology, host immune cell infiltration and cytokine production. The mouse model described here will allow examination of parasite pathogenic factors, as well as granting access to the increased availability of genetically modified mice and cell lines.

Many *Tv* strains have established symbioses with either the bacterium *Mycoplasma hominis* and/or double-strained RNA viruses (TVV) [35]. The strains we used for our analyses harbored the *M*. *hominis* symbiont, however, it is not known whether they also contained TVV. Future experiments, using this mice model and isogenic strains that differ only in the presence or absence of these symbionts, will help define the roles these symbionts play in establishing and maintaining *Tv* infection.

Persistent asymptomatic carrier state infection with *Tv* has been documented in men, whereas parasite clearance within 2 weeks has also been reported [73]. This male model of pathogenesis may allow specific factors of male trichomoniasis and infection outcome to be explored. Future experiments will be necessary to determine whether this model can be modified to establish and examine chronic infections in the mouse prostate and whether such infections are primarily asymptomatic or lead to inflammation and pathology. The ability to study chronic infections may allow better characterization of factors that contribute to adverse outcomes of infection. Information learned using the MUT model may also better inform design of vaginal models which would allow the contribution of the vaginal microbiota in *Tv* pathogenesis to be addressed.

## Supporting information

S1 Data Nluc signal is not altered in the absence of G418 selection. LSU 160 MA-Nluc (A), LSU 160 P-Nluc (B), 1103-Nluc (C) were passaged daily for up to 72 hrs in the absence of G418 selective pressure. Luminescence signal was assayed from 10^4^ parasites at 48 hrs and 72 hrs (X-axis) post-removal of G418 for each strain and found to have no significant difference in luminescence compared to their respective 0 hr control. Data shown are averages of luminescence signal with standard deviation and were carried out using 3 biological and 3 technical replicates for each strain.
(TIF)

S2 Data Nluc signal 4 d post-inoculation lies at the lower limit of Nluc detection. 10^8^ *Tv* parasites were introduced into the MUT, followed by excision of the MUT at 0 hr and 4 d post-inoculation (X-axis). The non-axial horizontal line denotes the lower limit of detection for the nanoluciferase assay. Data shown are averages of luminescence/μg protein of the sample with standard deviation using 3 mice per timepoint.
(TIF)

S3 Data Numerical values of luminescence in different Nluc-expressing *Tv* strains displayed in Fig 2. (XLSX)

S4 Data Numerical values for parasite burden displayed in Fig 3. (XLSX)

S5 Data Numerical values for *Tv* found within the murine MUT tissue displayed in Fig 4. (XLSX)

S6 Data Numerical values for viable parasites recovered from mouse MUT tissue displayed in Fig 5. (XLSX)

S7 Data Numerical values for parasites adhering to MUT tissue displayed in Fig 6. (XLSX)

S8 Data Numerical values for parasites adhering to MUT tissue displayed in Fig 7. (XLSX)
