# Supplementary figures and images for: Trichomonas vaginalis adherence phenotypes and extracellular vesicles impact parasite survival in a novel in vivo model of pathogenesis
Source: PLoS Negl Trop Dis. 2023 Oct 23;17(10):e0011693. doi: 10.1371/journal.pntd.0011693 (PMC10621976; doi:10.1371/journal.pntd.0011693)

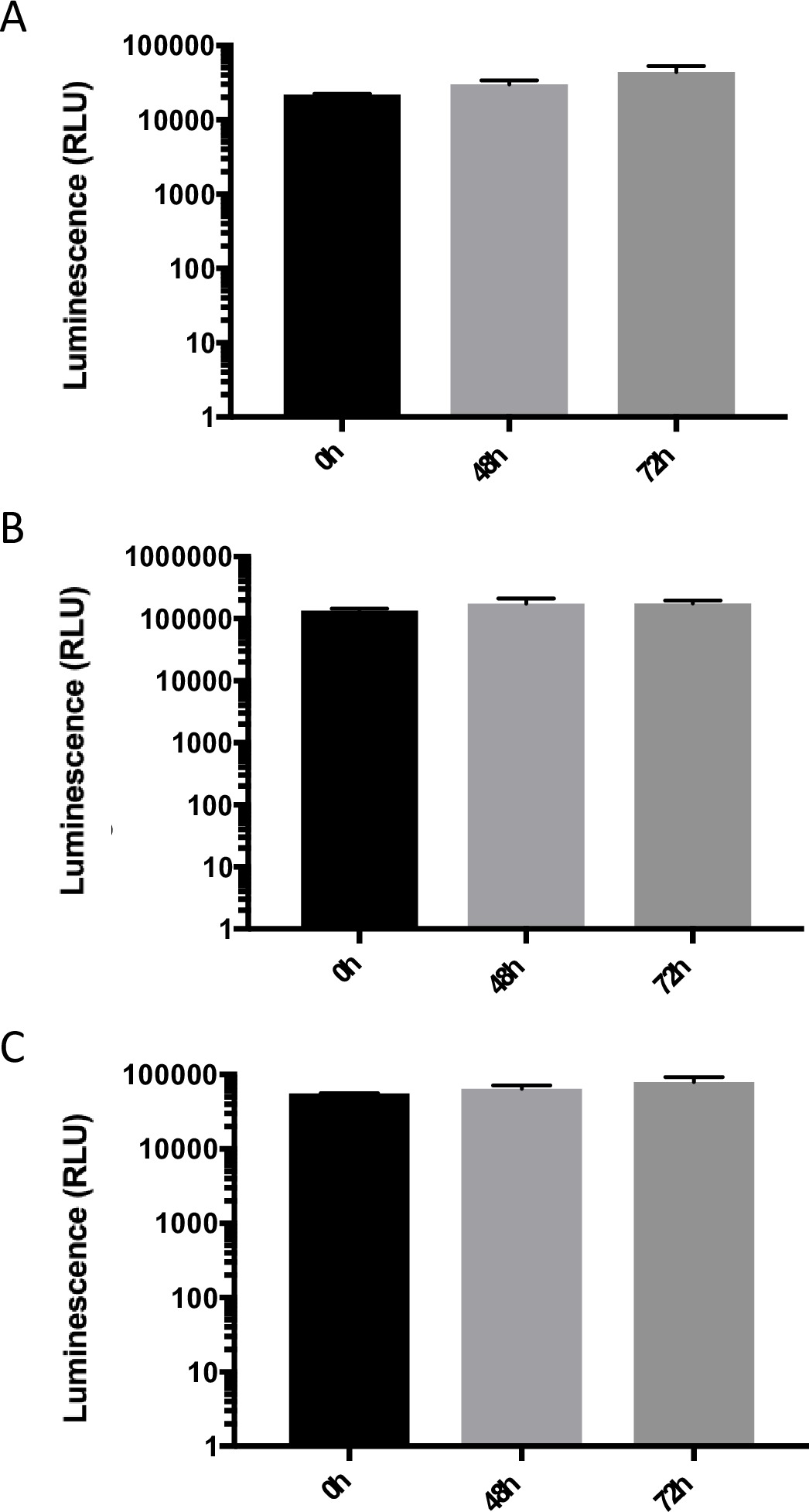

Supplement: S1 Data — LSU 160 MA-Nluc (A), LSU 160 P-Nluc (B), 1103-Nluc (C) were passaged daily for up to 72 hrs in the absence of G418 selective pressure. Luminescence signal was assayed from 104 parasites at 48 hrs and 72 hrs (X-axis) post-removal of G418 for each strain and found to have no significant difference in luminescence compared to their respective 0 hr control. Data shown are averages of luminescence signal with standard deviation and were carried out using 3 biological and 3 technical replicates for each strain. (TIF) [file pntd.0011693.s001.tif]

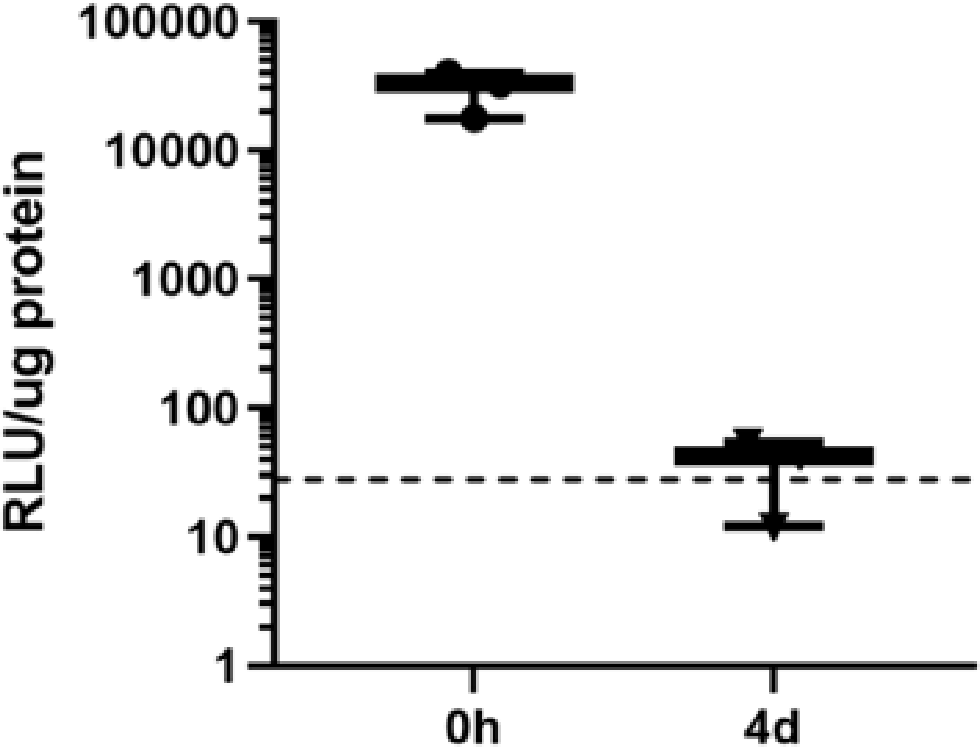

Supplement: S2 Data — 108 Tv parasites were introduced into the MUT, followed by excision of the MUT at 0 hr and 4 d post-inoculation (X-axis). The non-axial horizontal line denotes the lower limit of detection for the nanoluciferase assay. Data shown are averages of luminescence/μg protein of the sample with standard deviation using 3 mice per timepoint. (TIF) [file pntd.0011693.s002.tif]
